# Interleukin-23 and Th17 Cells in the Control of Gut Inflammation

**DOI:** 10.1155/2009/297645

**Published:** 2009-05-27

**Authors:** Ivan Monteleone, Francesco Pallone, Giovanni Monteleone

**Affiliations:** Department of Internal Medicine, University “Tor Vergata” of Rome, 00133 Rome, Italy

## Abstract

Crohn's Disease and Ulcerative Colitis, the major forms of inflammatory bowel diseases (IBDs) in humans, have been traditionally associated with exaggerated and poorly controlled T helper (Th) type 1 or Th2 cell response, respectively. More recent studies have, however, shown that IBDs are also characterized by a sustained production of cytokines made by a distinct lineage of Th cells, termed Th17 cells. The demonstration that Th17-related cytokines cause pathology in many organs, including the gut, and that expansion and maintenance of Th17 cell responses require the activity of IL-23, a cytokine made in excess in the gut of IBD patients has contributed to elucidate new pathways of intestinal tissue damage as well as to design new therapeutic strategies. In this review, we discuss the available data supporting the role of the IL-23/Th17 axis in the modulation of intestinal tissue inflammation.

## 1. Introduction

Crohn's disease (CD) and ulcerative colitis (UC) are the major forms of inflammatory bowel diseases (IBDs) in humans. The etiology of IBD is still unknown, but evidence has been accumulated to indicate that environmental and genetic factors contribute to promote an immunopathologic process leading to chronic inflammation [1]. Studies of experimental models of IBD and clinical observations also suggest that CD4+ T-cells play a major role in initiating and shaping the pathologic response in IBD. Consistently, T-cell-directed therapies have been employed with some clinical success in IBD patients [1–3]. 

The ability of CD4+ T-cells to alter the magnitude and outcome of the intestinal tissue-damaging inflammatory responses is mostly dependent on the production of distinct profiles of cytokines [4]. Traditionally, CD lesion has been associated with a predominant activation of T helper type 1 (Th1)-lymphocytes that produce large quantities of interferon (IFN)-*γ* under the stimulus of interleukin (IL)-12 [5–7]. By contrast, the UC lesion was believed to be driven by Th2-cytokines, such as IL-4 and IL-13 [8, 9]. More recently, studies from several laboratories had led to the identification of more complex networks of cytokine interactions in IBD tissue, thus shedding light into the role of a distinct subset of Th cells, namely, Th17 cells, in the pathogenesis of colitis [10–13]. In this paper, we will review the available data supporting the involvement of Th17 cells in the modulation of gut inflammation.

## 2. Th17 Cell Induction and Functions

Th17 cells have derived the name by their ability to produce IL-17, also termed IL-17A. Additional cytokines produced by Th17 cells include IL-17F, IL-21, IL-22, TNF-*α*, and IL-6 [14]. While Th1 and Th2 cell differentiations depend on their respective effector cytokines (i.e., IFN-*γ* and IL-4, resp.), induction of Th17 cells does not require IL-17A [14]. By contrast, Th17 cell development requires the activity of the transcription factors retinoic acid-related orphan receptor (ROR) *γ*t and ROR*α*, and RUNX1, and is driven by TGF-*β*1 [14–16]. Th1 and Th2-related cytokines inhibit Th17 cell differentiation, while IL-17 is not able to suppress Th1 and Th2 cells or does so very weakly [17]. Therefore, suppression of IFN-*γ* and IL-4 represents a way by which TGF-*β*1 could promote Th17 cell differentiation, even though TGF-*β*1-driven Th17 cell development can also occur in the absence of IFN-*γ* and IL-4 [18, 19]. The effects of TGF-*β*1 on Th17 cell differentiation are concentration dependent; low doses induce Th17 cells, while high doses inhibit Th17 cell development and promote regulatory T-cells (Tregs) [20]. Importantly, Tregs can convert into inflammatory Th17 cells in the presence of inflammatory cytokines such as IL-6 [21]. Since Th17 cells appear to be resistant to the Tregs-mediated immunosuppression [22], it is likely that during chronic inflammatory processes, such as IBD, Tregs may augment rather than suppressing Th17-mediated immune responses. 

 Th17 differentiation can be induced by IL-21 in IL-6-deficient mice [23]. These observations and the demonstration that IL-21 is made by T-cells in response to IL-6 clearly indicate that IL-21 acts downstream of IL-6 in Th17 cell generation. Importantly, IL-21 stimulates Th17 cells to produce IL-21, thus triggering an autocrine loop that amplifies Th17 cell responses [24]. IL-21 enhances the expression of IL-23 receptor (IL-23R) in Th17 cells, through a process that is dependent on Stat3 and ROR*γ*t, making these cells responsive to IL-23 [25]. Since IL-23 facilitates the expansion and/or stabilization of the Th17 phenotype, these data suggest a mechanism by which IL-21 could contribute to amplify Th17-cell responses. Th17 cell responses are also sustained by IL-1 [26]. 

Th17 cell-derived cytokines are supposed to play an important role in the protection of the host against various bacteria and fungi, particularly at mucosal surfaces, given their ability to enhance the recruitment and facilitate the activation of neutrophils and stimulate the production of defensins by epithelial cells [27, 28]. In line with this it was shown that Th17 cells are constitutively present in the human and mouse intestinal mucosa [29]. Studies in mice have also shown that, in the gut, commensal bacteria drive the differentiation of Th17 cells through a mechanism which is strictly dependent on the production of adenosine 5′-triphosphate (ATP) [29]. Additionally, it was shown that stimulation of dendritic cells with toll-like receptor (TLR) ligands induces synthesis of cytokines that promoted differentiation of IL-17 producing CD4+ T-cells [30]. Similarly, stimulation of dendritic cells with bacterial-derived ligands for NOD-2 results in enhanced IL-17 production by human memory T-cells, through an IL-1 and IL-23-dependent mechanism [31]. It has been also demonstrated that Th17 cells can be induced independent of TLR activation. For instance, engagement of the C-type lectin dectin-1 with curdlan stimulates dendritic cells to secrete IL-6, TNF-*α*, and IL-23 thus promoting the differentiation of Th17 cells [32]. IL-23 is absolutely required for providing Th17 cells a pathogenic phenotype. In fact, in the absence of IL-23, Th17 cells may have regulatory functions that correlate, in part, with their ability to produce IL-10 [33, 34]. 

Th17-related cytokines can trigger several inflammatory pathways due to their ability to enhance the synthesis of inflammatory cytokines (e.g., IL-1, IL-6, TNF-*α*, GM-CSF), chemokines (e.g., IL-8, CXCL1, CXCL8, monocyte chemoattractant protein-1, monocyte-inhibitor protein (MIP)-3*α*), cyclooxygenase-2, and tissue-degrading matrix metalloproteinases (MMPs) [27, 35].

## 3. Expression of Th17 Cytokines in Human IBD

In 2003, Fujino et al. showed an increased number of IL-17-producing cells in the inflamed gut of patients with CD and patients with UC in comparison to normal and disease controls [12]. By immunohistochemistry, it was shown that, in active UC, IL-17-expressing cells were located mainly within the lamina propria, while in active CD, these cells were scattered throughout the submucosa and muscularis propria. Major sources of IL-17 were CD3+ T-cells and CD68+ cells. In line with this, it was shown that RNA transcripts for IL-17A and IL-17F were upregulated in the inflamed mucosa of UC patients and CD patients [12, 36]. More recently Annuziato et al. demonstrated that the number of IL-17-producing T-cells is higher in CD than in normal gut mucosa, and that some of these cells produce also IFN-*γ* [37]. Stimulation of these cells with IL-12-enhanced expression of Th1-related markers (i.e., T-bet and IFN-*γ*) and downregulated ROR*γ*t and IL-17, thus indicating that IL-17-secreting T-cells can be induced to differentiate in fullypolarized Th1 cells [37]. There is also evidence that treatment of intestinal lymphocytes with IL-23 enhances either IL-17A or IFN-*γ* in UC or CD, respectively [38]. 

The inflamed mucosa of IBD patients contains high levels of other Th17-related cytokines. Indeed we have previously shown that in both CD and UC tissues there is enhanced production of IL-21, a cytokine that is capable of expanding the ongoing Th1 cell response in CD [39], stimulating gut fibroblasts to secrete MMPs [40], and inducing colonic epithelial cells to produce the chemokine MIP-3*α* [41]. IL-22 is also highly expressed in mucosal samples of patients with active CD and to a lesser degree of patients with UC [42]. Like other Th17 members, IL-22 stimulates colonic fibroblasts to make inflammatory cytokines (e.g., IL-6, IL-8, IL-11, and leukemia inhibitory factor), chemokines, and MMPs [42]. Moreover, IL-22 enhances the expression of TNF-*α*, IL-8, and *β*-defensin [43]. 

Upregulation of Th17-related cytokines does not however represent a specific hallmark of IBD, as increased levels of IL-17A and other Th17-markers have been seen in patients with rheumatoid arthritis, multiple sclerosis, and psoriasis [44–46]. Moreover, studies in murine models of these pathologies strongly suggest that Th17 cells play a pivotal role in propagating tissue-damaging immune responses [47–49].

## 4. Role of Th17 Cells in the Pathogenesis of Experimental Colitis

By using IL-17 receptor A (IL-17RA) knockout mice, Zhang et al. showed that IL-17 is necessary for the development of colitis induced by intrarectal administration of trinitrobenzenesulfonic (TNBS) acid [50]. Consistently, administration of IL-17RA IgG1 fusion protein in mice with TNBS-colitis significantly attenuated colonic inflammation and prevented weight loss. In this context it is however noteworthy that IL-17RA mediates the activities of both IL-17A and IL-17F [51], making difficult to establish the exact contribution of these cytokines in the pathogenesis of TNBS-colitis. Studies in other models of colitis, such as the dextran sulfate sodium (DSS)-induced colitis, showed that IL-17F deficiency results in reduced colitis, whereas IL-17A-null mice develop more severe disease [52]. 

**Figure 1:**
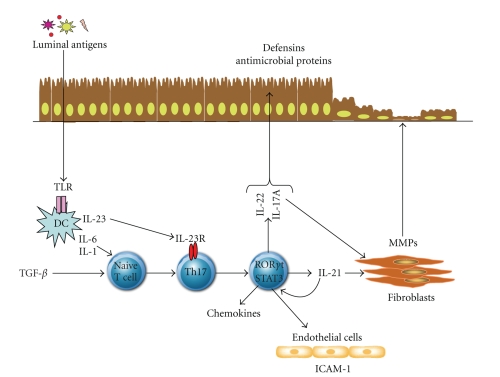
Role of Th17 cells in the modulation of intestinal inflammation. Th17 cells differentiate from naïve T lymphocytes under the stimulus of TGF-*β*, IL-6, and IL-1, while the dendritic cells derived IL-23 and the Th17 cell-derived IL-21 contribute to maintain/expand Th17 cell populations. Th17-derived cytokines, such as IL-17A, IL-21, and IL-22, promote the recruitment of inflammatory cells in the intestinal lamina propria, due to their ability to enhance the synthesis of chemoattractants and adhesion molecules (e.g., ICAM-1) by epithelial and endothelial cells, respectively. IL-17A, IL-21, and IL-22 stimulate fibroblasts to make matrix metalloproteinases, a family of enzymes that could contribute to the tissue damage and remodeling occurring in IBD. IL-17A and IL-22 also stimulate the synthesis of antibacterial proteins, including defensins, by epithelial cells. DC; Dendritic cells; TGF*β*; transforming growth factor-*β*; MMPs; matrix metalloproteinases; ICAM-1; intercellular adhesion molecule-1.

Th17 cells seem to be involved in the pathogenesis of colitis induced by transfer of a cecal bacterial antigen-specific C3H/HeJBir (C3Bir) CD4 (+) T-cell line to C3H/HeSnJ SCID mice [13] (see Figure 1). In this model, colitis associated with enhanced production of IL-17, and adoptive transfer of IL-17-secreting T-cells to SCID recipients resulted in a marked gut inflammation, as compared to that caused by transfer of Th1 cells. Administration of mice with a monoclonal anti-IL-23p19 prevented and treated active colitis, downregulated the synthesis of a broad array of inflammatory cytokines and chemokines in the colon, and promoted apoptosis of colitogenic Th17 cells. By using a novel model of CD8+ T-cell-dependent colitis, Tajima et al. have recently shown that a single adoptive transfer of naïve CD8+ T-cells into syngeneic RAG-deficient mice was followed by rapid spontaneous proliferation of these cells in the mesenteric lymph nodes and severe colitis [53]. Analysis of cytokine-secreting CD8+ T-cells in the mesenteric lymph nodes showed the existence of IL-17 and IFN-*γ*-double-positive cells. Notably, adoptive transfer of naïve CD8+ T-cells derived from either IL-17- or IFN-*γ*-knockout mice associated with a remarkably less severe colitis, raising the intriguing possibility that IL-17 and IFN-*γ* can cooperate to cause pathology in this model of colitis. Consistent with these findings is our demonstration that IL-21-null mice are largely protected against the development of DSS-colitis and TNBS-relapsing colitis [54]. This protection was associated with a reduced colonic expression of several Th17- and Th1-related genes. Additionally, blockade of IL-21 activity with a specific IL-21R-fusion protein reduced intestinal inflammation and Th17 response during the course of DSS colitis [54]. 

Taken together these findings suggest that Th17 cytokines contribute to orchestrate the T-cell response that causes tissue damage in the gut.

## 5. IL-23 and Gut Inflammation

IL-23 is a heterodimeric cytokine composed by the specific p19 subunit and the p40 chain that is shared with IL-12 [55]. The synthesis of the p40 subunit and the functional heterodimeric IL-23 is enhanced in the gut of CD patients [5, 56]. In a recent study, kamada et al. showed that IL-23 is made preferentially by a subset of mucosal cells expressing both macrophage (i.e., CD14, CD33, CD68) and dendritic cell markers (i.e., CD205, CD209) and present in large numbers in CD tissue [57]. These cells induce lamina propria mononuclear cells to make IFN-*γ* rather than IL-17 production through an IL-23 and TNF-alpha-dependent mechanism [57]. This substantiates further the concept that, in CD, IL-23 may expand the ongoing Th1 cell response In line with these data, a neutralizing p40 monoclonal antibody has shown clinical efficacy in patients with moderate and severe CDs [58]. If the therapeutic effect of this novel reagent is due to the neutralization of IL-12 and/or IL-23 remains to be ascertained. However, studies conducted in various animal models of colitis would seem to indicate that IL-23 is more pathogenic than IL-12 in the gut. For instance, by backcrossing IL-10-deficient mice with mice lacking IL-12p35 or IL-23 p19, Yen et al. showed that IL-23 was essential for manifestation of chronic intestinal inflammation, whereas IL-12 was not [59]. CD4+ T-cells from IL-10/p19-knockout mice still produced large amounts of IFN-*γ*, thus indicating that Th1 responses developed normally in the absence of IL-23, but disease manifestations required the presence of IL-23. Moreover, administration of exogenous IL-23 in RAG mice reconstituted with naïve CD4+ T-cells caused a more severe colitis that was associated with enhanced production of IL-6 and IL-17 and preventable by treatment of mice with a blocking IL-6 or IL-17 antibody. Although in this model, IL-6 and IL-17 were made by memory T-cells, there is no doubt that some of the pathogenic functions of IL-23 in the gut are mediated by non-T-cell populations. This was first shown by Powrie et al. who analyzed the effect of an agonistic anti-CD40 antibody in RAG mice lacking IL-23p19 or IL-12p35 [60]. Administration of anti-CD40 caused a systemic and local inflammation characterized by wasting disease, splenomegaly, increases in serum proinflammatory cytokines, and colitis. It was shown that the systemic inflammatory response and the elevated concentrations of proinflammatory cytokines in the serum were driven by IL-12 while the local intestinal inflammation and production of IL-17 in the intestine were controlled by IL-23. More recently, using the T-cell transfer model of colitis, the same group has shown that IL-17-deficient T-cells are not impaired in their ability to induce colitis in RAG mice [61]. Protection of colitis seen in RAG mice lacking IL-23 was associated with no decrease in the levels of IL-17, as well as lack of IL-23 did not significantly affect the relative amounts of the Th17-specific factor ROR*γ*t in the colon. Consistent with this study, Noguchi et al. showed that transfer of naïve CD4+ T-cells prepared from IL-17-knockout mice induced severe colitis in RAG mice [62]. Together, these data indicate that Th17 cell responses are not specifically impaired in the intestine of IL-23-deficient mice and that IL-23-mediated colitis is not strictly dependent on IL-17 production. 

A more detailed analysis of the mechanisms underling the IL-23-dependent pathologies revealed that IL-23 can facilitate colitis not only via direct effects on inflammatory mediators but also indirectly by counteracting regulatory mechanisms. Indeed, protection of colitis seen after transfer of naïve T-cells to RAG mice lacking IL-23 was associated with an increase in the frequency of Foxp3-expressing T regulatory cells in the intestine [61]. Upon naïve T-cell transfer, administration of mice with either an antagonistic IL-10R antibody or a blocking TGF-*β* antibody increased colonic inflammation compared to untreated controls. Moreover naïve T-cells isolated from transgenic mice expressing a dominant-negative form of TGF-*β* receptor II and unable to respond to TGF-*β* induced significant colitis when transferred to IL-23-deficient RAG mice [61]. High levels of IFN-*γ*, but not IL-17, were seen in colitic mice, thus suggesting that IFN-*γ* might drive the chronic intestinal inflammation in this setting. Notably, transfer of Foxp3-deficient T-cells to IL-23-deficient RAG mice caused severe colitis, indicating that IL-23 is not essential to the pathogenesis of intestinal inflammation, if counterregulatory mechanisms are defective or absent [61]. These later findings well fit with the notion that the requirement of IL-23 for the initiation and progress of gut inflammation varies depending on the model. In fact, acute colitis induced by TNBS is driven by IL-12 and negatively regulated by IL-23 [63].

## 6. Conclusions

The recent discovery that IL-23 and Th17 cells play a major role in the development of chronic intestinal inflammation in mouse models of IBD together with the identification of IL-23R gene polymorphisms that influence IBD susceptibility [64] has provided a new picture of the way the local immune response can promote intestinal tissue damage. These new data suggest that, at least in theory, the IL-23/Th17 axis could be a promising target for suppressing inflammation in IBD. This is in line with the demonstration that blockade of cytokines that positively regulate Th17 cell polarization can dampen colitis. For instance, a monoclonal antibody targeting IL-6R has been successfully used in patients with active CD [65]. Both in vitro and in vivo data showed that anti-IL-6 treatment enhanced mucosal T-cell apoptosis. However, given the critical role of IL-6 in the differentiation of Th17 cells [66], it is tempting to speculate that the beneficial effect of anti-IL-6R may in part rely on the control of Th17 cell responses. Nonetheless, some important issues remain to be resolved. For example, emerging evidence suggests that Th17 cell biological effects are strictly context-dependent, and that Th17 cells can promote either protective or pathogenic responses depending on additional factors present within the mucosal milieu. So, further experimentation is needed to identify such factors. Mechanisms that trigger and regulate Th17 cell response in human IBD remain to be elucidated. Similarly it is unknown whether and how these cells interact in vivo with other regulatory and effector mucosal T-cell subsets. It is also unclear whether the various Th17 cytokines make qualititatively and quantitatively different contributions to the initiation and progress of gut inflammation or whether they are redundant in their ability to modulate specific inflammatory pathways. It remains also to be clarified why mice lacking IL-23 or specific Th17-related genes are differently susceptible to specific forms of experimental colitis. As pointed out above, Th17-cytokines are constitutively produced in the normal human and mouse gut, raising the possibility that these molecules may be involved in the maintenance of immunological homeostasis and/or in the control of specific inflammatory pathways. If this is the case, blocking Th17-cytokines could have deleterious rather than beneficial effects for the host. 

Finally, experimental studies are still necessary to ascertain whether prolonged treatment with biological compounds that interfere with Th17 cell functions can enhance the risk of infections and cancer, given that Th17 cytokines mediate host-defensive mechanisms to bacteria and fungi and exert antitumor activity.
